# Everybody's Page

**Published:** 1889-10-05

**Authors:** 


### Everybody's Page.

The last sanitary report of the kingdom of Norway showed that in its 43 hospitals there were treated 10,020 persons, of whom 941 died, showing a mortality of about ten per cent. There were 2,009 persons in its asylums.

Latin has not gone out of date so completely as some people would have us believe. The Hungarian pharmacopeia or "Magyar Gyogyszerkonyre," was published both in Hungarian and Latin, for the benefit of those who do not understand the former tongue.

The custom, now so prevalent, of grocers selling drugs is only a return to a very old one. Formerly the apothecaries and the grocers formed one body, but after a time the Society of Apothecaries separated from their old companions on account of the "sophistications" introduced by the latter into their wares, and did their best to introduce pure drugs.

A French writer has come to the conclusion that the pianist is the most wonderful creature in the world. His cerebral operations—reading two lines of music, interpreting each by means of his fingers, putting in appropriate expression, varying the time in infinitesimal degrees as his idea of the piece demands—these show greater complexity than is demanded in any other occupation.

One of the earliest museums in England, according to Prof. Flower, was that formed by John Tradescant at South Lambeth. It included many curiosities which the authorities of the British Museum would be delighted to obtain, for example, the Dragon's Egge from Turkie, two feathers of a Phœnix Tayle, and the Claw of the bird Rock, who, as authors report, is able to trusse an elephant. What biologist would not long to study these?

Washing and boiling is, as a rule, a sufficient method of disinfecting the clothes worn by the sick, but the infectious matter is thus only transferred to the water used in the process, and from the steaming tub a new danger may arise. Many cases have been known in which those who have washed the linen of cholera patients have contracted the disease, and in one case a number of persons caught typhoid fever from drinking the water of a well into which, by some accident, the suds in which the linen of a typhoid patient was washed had been introduced.

Dr. C. M. Jessop has been running counter to all popular notions on the subject of beef-tea. Clear beef-tea he regards as worthless, and he thinks that in beef-tea worthy of the name there should be no refuse or remainder. The meat, well chopped, should be placed in a digester and boiled for three hours, during which time it is frequently stirred and pounded with a wooden masher. At the end of this time it is to be passed through a colander to make it of a smooth consistency, but nothing is to be thrown away. The patient is to eat every part of the beef, but in a liquid instead of a solid condition.

The steps by which memory is lost have been analysed by M. Nivelet in a work on what he calls auto-psychologie. First go the names of persons and places, then the common nouns and other words which are not, however, frequently employed in daily life. The ordinary phrases of conversation remain for a much longer time, though the power to spell the words in them is gone. Simultaneously with this loss of memory of common things and recent events, is a renewed recollection of childish days, old companions, phrases forgotten through all the years of manhood—a sort of palimpsest of childhood that reappears as the experiences of maturity are wiped away from the memory.

Mexico is not a bad place for babies. The import duty on drugs is so high there that only those who are fairly well off can afford to drug their children with soothing syrups and patent medicines of a similar order.

In the fifth year of the French republic—the first republic—Legrand d'Aussey, a Jesuit priest and a member of the Institute of France, declared the necessity of substituting cremation for earth-burial, and a prize of fifteen hundred francs was voted by the Institute to encourage the scientific study of the question.

Ambulances first appear in 1597, and military hospitals soon after. The French were the first to make provision for those wounded in war, and in 1630, in preparing for the campaign in Italy, Cardinal Richelieu organised an army medical service, and gave its chief the title of "Surgeon-Major of the Camps and Armies of the King."

For years German manufacturers bought tea that had suffered some accident which rendered it useless as a beverage from English bonded warehouses at a very low price, and of course, duty free, and extracted caffeine from it, which they sold at a good price to English chemists. It is only recently that English chemists have followed the example of their Teutonic kinsmen.

One of the difficulties experienced by those who strive to provide better homes for the working classes, is that the working classes grudge spending money on healthy homes. Some want to save all they can; others care more for the delights of the public-house, or the Sunday dinner, too solid to be wholesome, or clothing too gorgeous to be tasteful. They haven't any to spare for house-rent.

It is not generally supposed that the English have added much to the gaiety of nations, but they introduced into France the pleasing entertainment of the greased pole. The first time the game was played was in 1425, at the time when the English domination was most powerful on the other side of the Channel. The incident is reported in a contemporary work, the "Journal of a Parisian Bourgeois under Charles VII.," as having taken place on St. Giles's Day—that is, the first of September.

A cure for the bad habit of swearing is recorded in Mr. S. J. Holyoake's volume on "Self-Help a Hundred Years Ago." One Richard Bettany, of Dillorn, in Staffordshire, a collier by trade, was greatly given to the use of profane language. When some land about Dillorn was enclosed, Bettany was allotted a cottage and a plot of ground; but he was told that now that he possessed land he would be treated as a gentleman, and, therefore, if he swore he would be fined for it. His new importance, and an objection to paying a fine, had a beneficial effect, and he very soon left off his bad habit.

A curious accident which happened recently in Paris points out a possible danger in the wearing of combs and bracelets of celluloid. A little girl sat down before the fire to prepare her lessons. Her hair was kept back by what used to be called a "crop-comb," a semi-circular comb of celluloid. As her head was bent forward to the fire, this became warm, and suddenly burst into flames. The child's hair was partly burned off, and the skin of the head was so injured that several months after, though the burn was healed, the cicatrix formed a white patch on which no hair would grow. The burning point of celluloid is about 180deg., and the comb worn by the girl had attained that heat as it was held before the fire.

